# Role of Mitofusin 2 in the Renal Stress Response

**DOI:** 10.1371/journal.pone.0031074

**Published:** 2012-01-26

**Authors:** Jonathan M. Gall, Zhiyong Wang, Marc Liesa, Anthony Molina, Andrea Havasi, John H. Schwartz, Orian Shirihai, Steven C. Borkan, Ramon G. B. Bonegio

**Affiliations:** 1 Renal Section, Boston Medical Center, Boston, Massachusetts, United States of America; 2 The Obesity Center, Boston Medical Center, Boston, Massachusetts, United States of America; Instituto de Química - Universidade de São Paulo, Brazil

## Abstract

The role of mitofusin 2 (MFN2), a key regulator of mitochondrial morphology and function in the renal stress response is unknown. To assess its role, the MFN2 floxed gene was conditionally deleted in the kidney of mice (MFN2 cKO) by Pax2 promoter driven Cre expression (Pax2Cre). MFN2 cKO caused severe mitochondrial fragmentation in renal epithelial cells that are critical for normal kidney tubular function. However, despite a small (20%) decrease in nephron number, newborn cKO pups had organ or tubular function that did not differ from littermate Cre-negative pups. MFN2 deficiency in proximal tubule epithelial cells in primary culture induced mitochondrial fragmentation but did not significantly alter ATP turnover, maximal mitochondrial oxidative reserve capacity, or the low level of oxygen consumption during cyanide exposure. MFN2 deficiency also did not increase apoptosis of tubule epithelial cells under non-stress conditions. In contrast, metabolic stress caused by ATP depletion exacerbated mitochondrial outer membrane injury and increased apoptosis by 80% in MFN2 deficient *vs.* control cells. Despite similar stress-induced Bax 6A7 epitope exposure in MFN2 deficient and control cells, MFN2 deficiency significantly increased mitochondrial Bax accumulation and was associated with greater release of both apoptosis inducing factor and cytochrome c. In conclusion, MFN2 deficiency in the kidney causes mitochondrial fragmentation but does not affect kidney or tubular function during development or under non-stress conditions. However, MFN2 deficiency exacerbates renal epithelial cell injury by promoting Bax-mediated mitochondrial outer membrane injury and apoptosis.

## Introduction

Mitochondria are dynamic structures with multiple roles in cell homeostasis and death. Mitochondrial re-modeling is continuously regulated by opposing fission and fusion events that determine whether mitochondria appear long and filamentous (fused), or short and punctate (fragmented). A limited number of proteins regulate fission and fusion. Mitofusin 1 and 2 (MFN1, MFN2), both located on the outer mitochondrial membrane and Opa1, located on the inner membrane (reviewed in [1]) and are key regulators of mitochondrial fusion.

Dynamic organelle re-modeling ensures retention of intact copies of mitochondrial DNA [2], [3], [4], facilitates mitochondrial division into daughter cells [5], [6] and prepares unhealthy mitochondria for breakdown and recycling by mitophagy [7]. Mitochondrial fission and fusion are critical to the function of highly polarized cells (e.g., neurons and epithelial cells) [5], [8], [9] by promoting the intracellular transport of these organelles to their required intracellular locations. In select cell lines, the morphologic state of mitochondria determines baseline organelle function. For example, elimination of MFN2 in fibroblasts, L6E9 myotubes, or Opa1 in mouse embryonic fibroblasts increases mitochondrial fission, thereby decreasing mitochondrial membrane potential, oxygen consumption, glucose and palmitate oxidation and respiratory complex activity [10], [11], [12].

Recent evidence suggests that mitochondrial re-modeling is critical for normal organ development and function and mediates susceptibility to stress. Elimination of the dynamin-1-like (Dnm1l) gene, critical for mitochondrial fragmentation, causes death of python embryos during gestation. Heterozygous python hearts showed marked ATP depletion and reduced levels of key mitochondrial enzyme complexes associated with a severe cardiomyopathy [13]. Most mice lacking pro-fusion proteins (MFN1, MFN2, or Opa1), do not survive past mid-gestation [14], [15]. Death may be due to placental malfunction caused by MFN2-mediated failure of the trophoblast giant cell layer [14]. Surviving MFN2 knockout pups develop fatal cerebellar defect associated with unsteady gait, difficulty feeding and early failure to thrive [8]. In contrast, the role of mitochondrial remodeling in kidney development is unknown.

Diverse insults cause mitochondrial fragmentation in numerous organisms [16], [17], [18], [19] and in diverse cell types [17], [20], [21], [22], suggesting that mitochondrial morphology and survival are inter-related during stress. Furthermore, manipulation of mitochondrial dynamics markedly alters the susceptibility of non-renal cells to stress. Pro-apoptotic BCL family proteins not only mediate cell survival, but also regulate mitochondrial fusion and fragmentation (reviewed in [23], [24]). Of the BCL proteins, abundant evidence supports a role for Bax, previously considered a cell death protein, in regulating mitochondrial fusion and fragmentation. In non-stressed cells, inactive Bax promotes mitochondrial fusion [25]. In cells subjected to stress however, activated Bax promotes fragmentation via its effects on Drp1 [26] as well as MFN1 and 2 [27]. We and others have shown that Bax is a critical mediator of renal epithelial cell apoptosis after stress [28], [29], [30], [31], [32], [33], suggesting that mitochondrial remodeling and Bax may be intertwined in both renal development and organ dysfunction after stress.

Remarkably few studies have examined the role of mitochondrial re-modeling in the kidney. This is surprising, since normal nephron and organ function is highly dependent upon polarized, mitochondrial rich epithelial cells. During stress, epithelial cells in the proximal tubule rapidly undergo dramatic lethal and sublethal changes that contribute to organ failure ([34], [35], [36] reviewed in [37]). Importantly, the morphologic state of mitochondria could determine their susceptibility to injury. A few studies suggest that filamentous mitochondria are resistant to apoptotic cell death, whereas fragmented organelles are highly sensitive to apoptogenic stress [38], [39]. The reason for this marked difference in cell sensitivity to death signals in elongated *vs.* punctate mitochondria is unclear.

In the present study, we hypothesized that mitochondrial fusion is critical for renal development and organ function at baseline and following stress. To test this hypothesis, deficiency of MFN2, a key mitochondrial fusion protein, was conditionally generated in the murine kidney *in vivo* and in proximal tubule epithelial cells *in vitro*. Surprisingly, we find that MFN2 is not required for kidney development or normal organ function but is essential for renal cell survival after metabolic stress that is characteristic of ischemic kidney disease.

## Results

### Breeding and Phenotype of MFN2 cKO mice

**Figure 1:**
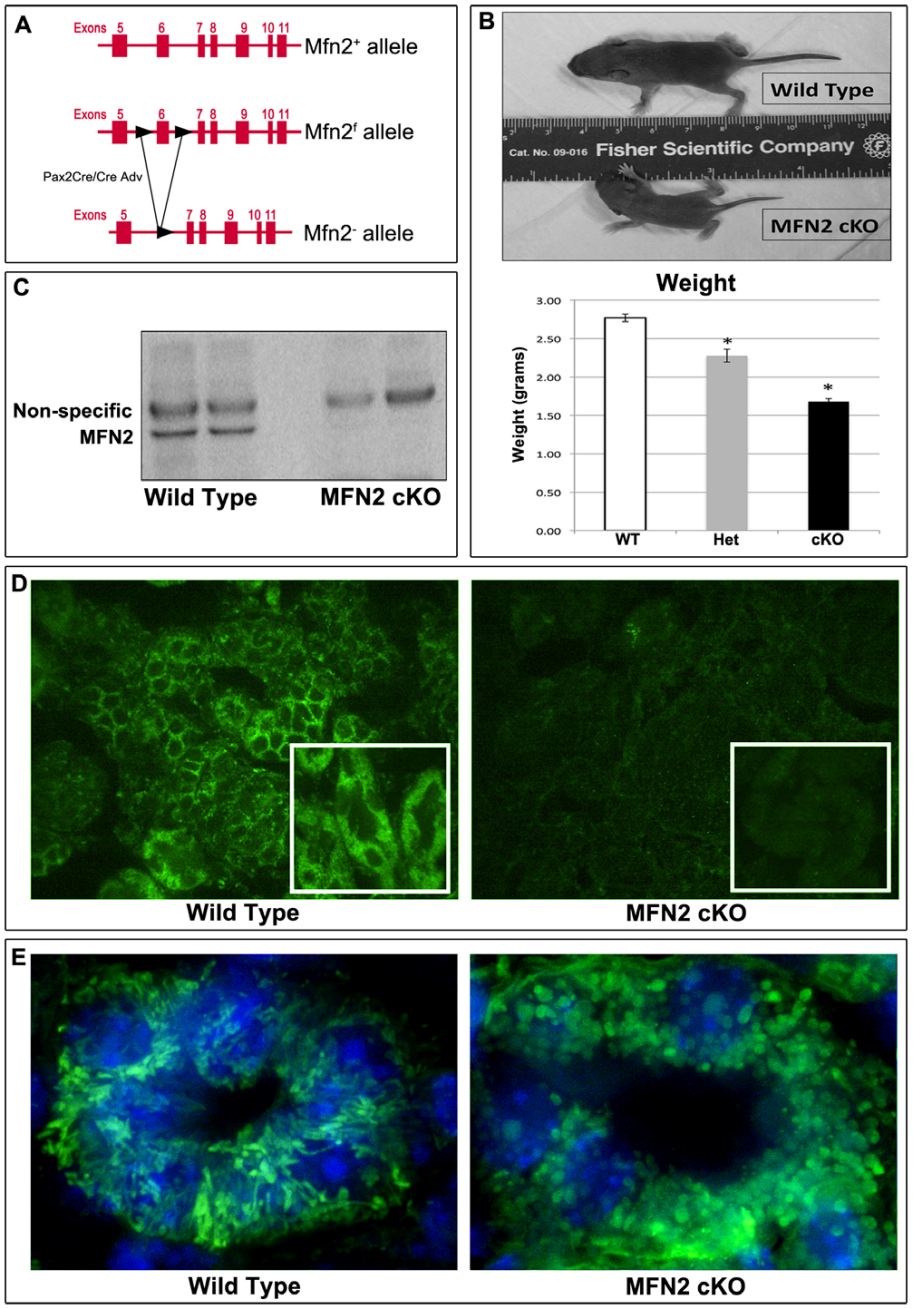
Generation and phenotype of MFN2 cKO mice. (**A**) Two LoxP sites flank exon 6 in the region coding for the canonical G-1 GTPase motif are shown. Cre recombinase excises exon 6 that causes a frame shift which precludes functional protein production. For *in vivo* experiments, animals were crossed with Pax2-Cre expressing mice creating MFN2 conditional knockouts (MFN2 cKO). (**B**) MFN2 cKO mice are significantly smaller than wild type littermate controls. * *P*<0.05 *vs. WW*; n≥8 per group. (**C**) MFN2 content in wild type and MFN2 cKO kidney homogenates. The MFN2-specific band is absent in homogenates harvested from MFN2 cKO mice. (**D**) MFN2 immunofluorescence staining in littermate wild type and MFN2 knockout kidney sections. MFN2 staining is absent in MFN2 cKO animals; 200× magnification (*inset*: 400× magnification). (**E**) Immunostaining for F_1_F_0_-ATPase, an intrinsic mitochondrial membrane protein, in wild type and MFN2 cKO kidney; mitochondria(*white arrows*) in tubule epithelia of MFN2 cKO mice appear fragmented and punctate compared to wild type littermates. Hoechst dye was used to stain tubule cell nuclei.

MFN2^f/f^ females were bred with Pax2-Cre^+^/MFN2^f/+^ males. The Pax2 promoter drives expression of Cre recombinase in all developing epithelial tissues of the kidney as well as the developing otocyst and midbrain-hindbrain boundary. In this study, Pax2-Cre^+^/MFN2^f/f^ animals are termed “MFN2 conditional knockouts” (MFN2 cKO; **Fig. 1A**). The mice were born in the expected Mendelian ratios. However, MFN2 cKO pups had difficulty maintaining an upright position and had small or absent milk spots, consistent with cerebellar dysfunction and feeding abnormality described in MFN2^f/f^ mice crossed with Meox2-Cre animals [8]. On day 4, MFN2 cKO mice weigh less than heterozygotes and controls (**Fig. 1B**). MFN2 cKO, but not Pax2-Cre^+^/MFN2^f/+^ or Pax2-Cre^−^ controls, died by day 6 post-partum. As a result, all kidneys from these animals were analyzed on post-natal day four.

### Pax2-Cre efficiently deletes MFN2 in kidney epithelial cells

MFN2, which is readily detectable in kidney from wild type neonatal mice, was undetectable in kidney homogenates of MFN2 cKO mice by immunoblot analysis (**Fig. 1C**) or immunostaining of kidney sections (**Fig. 1D**). Kidneys harvested from Pax2-Cre^+^/MFN2^f/+^ exhibited highly variable MFN2 expression (*data not shown*). MFN2 cKO mice had defective mitochondrial fusion in renal epithelial cells as evidenced by the presence of punctate, fragmented mitochondria while mitochondria of control mice appeared filamentous (**Fig. 1E**).

### MFN2 cKO mice have normal kidney histology and a low level of apoptosis

**Figure 2:**
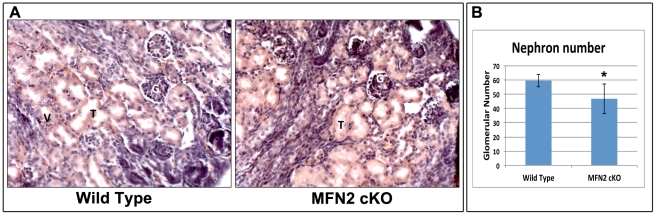
Effect of MFN2 conditional knockout on renal histology. (**A**) Wild type and MFN2 cKO kidney sections stained with hematoxylin and eosin; Tubules (**T**), glomeruli (**G**) or vasculature (**V**) appeared similar in both tissue sections. (**B**) Morphometric analysis of glomerular number in kidneys of wild type and MFN2 cKO littermates (n = 5/group). Data represent the mean and SD; * *P*<0.05.

**Figure 3:**
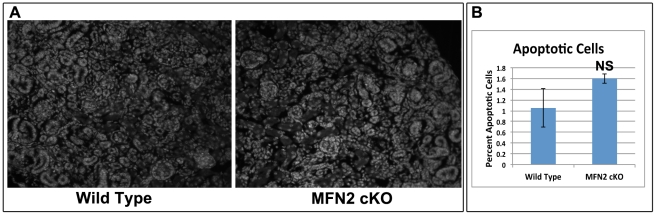
Effect of MFN2 conditional knockout on renal apoptosis. (**A**) Wide field fluorescent microscopy of kidney sections from wild type and MFN2 cKO four day old littermates stained with Hoechst dye injected intraperitoneally prior to sacrifice; remarkably few apoptotic cells were detected in either group. (**B**) Quantitative analysis of apoptotic cells in kidney sections of wild type and MFN2 cKO mice (n = 5/group). Data represent the mean and SD; * *P*>0.05.

Kidney sections obtained from four day old MFN2 cKO pups and control littermates were stained with hematoxylin and eosin. MFN2 cKO did not alter renal morphology and structure. In both groups, tubular, glomerular and vascular development was evident (**Fig. 2A**). However, compared to Cre-negative littermate controls, blinded morphometric analysis did reveal a small but statistically significant decrease (−20%) in the number of normal appearing nephrons in MFN2 cKO (**Fig. 2B**). A low level of apoptosis was detected at this developmental stage in kidney tissue of both MFN2 cKO and control littermates (**Fig. 3A**) and the number of Hoechst dye positive apoptotic cells detected in MFN2 cKO and wild type kidneys (1.6% vs. 1.1% respectively, *P* = 0.06) mice did not significantly differ (**Fig. 3B**).

### MFN2 does not cause gross changes in early postnatal renal function

**Figure 4:**
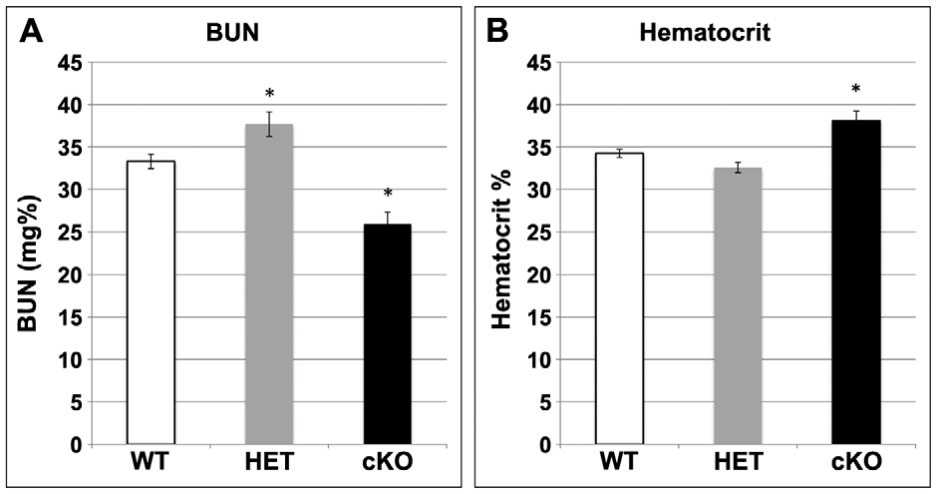
Effect of MFN2 conditional knockout on hematocrit and blood urea nitrogen (BUN). Hematocrit and BUN levels four days after birth. (**A**) Hematocrit was minimally increased in MFN2 cKO animals suggesting that they were volume depleted (**B**) BUN was slightly decreased in MFN2 cKO animals. (* *P*<0.05 *vs.* wild-type (WT); n = 8/group). Data represent the mean and SE; *P*<0.05.

Blood urea nitrogen (BUN) level was modestly decreased in MFN2 cKO animals compared to control (**Fig. 4A**), most likely a consequence of decreased dietary protein intake. In keeping with this finding, the hematocrits were comparable in Cre+/MFN2^f/+^ and Cre-negative controls, but elevated in MFN2 cKO pups that were noted to feed poorly and to be volume depleted (**Fig. 4B**). To exclude subtle tubular defects, the urine of MNF2 cKO mice was tested for specific gravity and pH as well as the abnormal presence of glucose and protein. Neither glucosuria nor proteinuria was detected in MFN2 cKO or control animals (**Table 1**). There was no difference observed in the specific gravity or pH of these two groups. Taken together, kidney and tubular function in normal and MFN2 cKO newborn mice appeared to be indistinguishable.

**Table 1:** Effect of MFN2 conditional knockout on tubular function in four day old mice.

	Wild Type (n = 8)	MFN2 cKO (n = 5)
**Glucose**	Negative	Negative
**Protein**	Trace - +	Trace - +
**Specific Gravity**	1.015–1.020	1.015–1.020
**pH**	5–6	6

MFN2 cKO did not result in overt urinary defects in either protein or glucose excretion, pH, or specific gravity.

### MFN2-deficiency induced mitochondrial fragmentation in cultured primary proximal tubule cells

**Figure 5:**
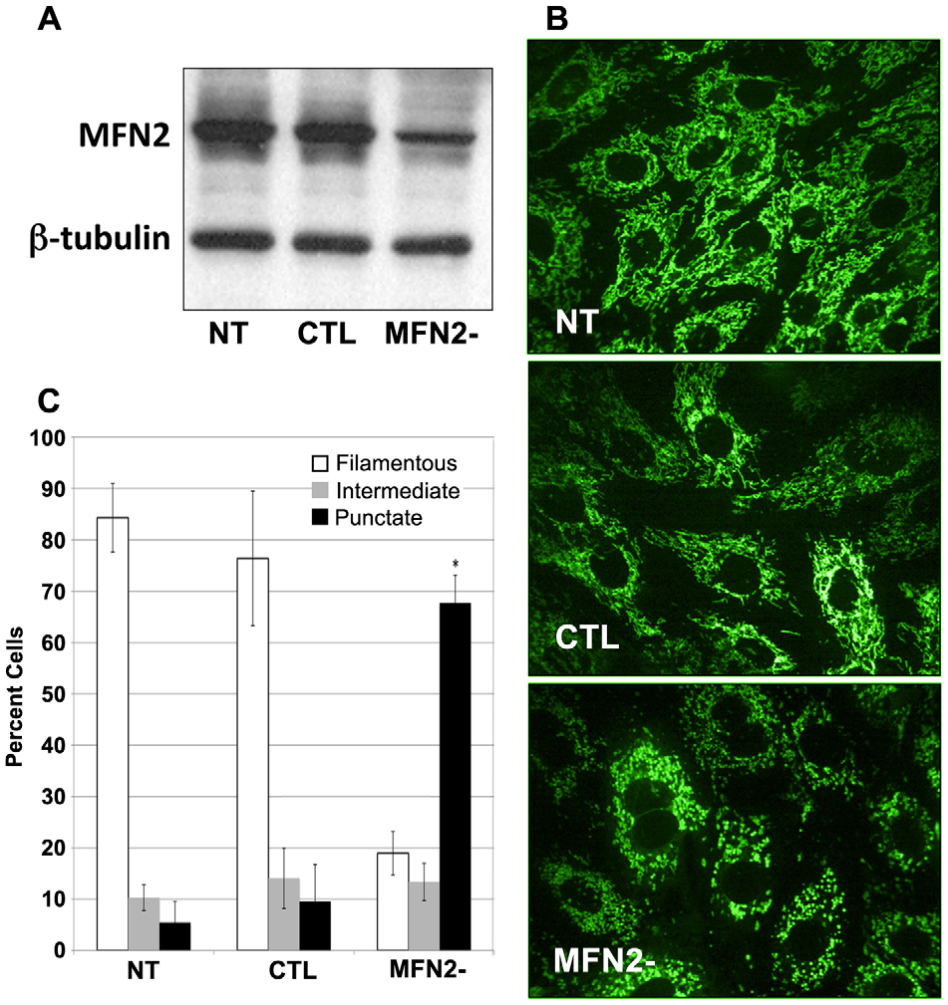
MFN2 deficiency causes fragmentation of mitochondria *in vitro*. (**A**) MFN2 content in MFN2^f/f^ primary cell cultures treated with Cre adenovirus (MFN2-), empty adenovirus vector (CTL), or no virus (no treatment or “NT”). (**B**) Mitochondrial morphology in primary proximal tubule epithelial cell cultures treated as described in (A) above and then stained with MitoTracker Green FM before visualization with a confocal microscope. (**C**) Percentage of primary cells with filamentous, intermediate, or punctate mitochondria assessed by a blinded reviewer after transduction with adenovirus as described above; * *P*<0.05 *vs.* either NT or CTL; data represent the mean and SE of three separate experiments; *P*<0.05.

The small body size of these pups precluded performing renal ischemia *in vivo*. To assess the susceptibility of cells lacking MFN2 to stress, proximal tubule cells were subjected to *in vitro* ATP-depletion using an established model. To delete MFN2 *in vitro*, primary cultures of proximal tubule cells harvested from MFN2^f/f^ mice were transduced with Cre-expressing adenovirus (MFN2-). Compared to non-transduced (NT) or control empty virus transduced (CTL) cells, CRE transduction reduced MFN2 expression by about 50–75% (**Fig. 5A**). Furthermore, CRE-mediated MFN2 reduction but not empty virus caused MitoTracker Green-stained mitochondria to appear punctate indicating that mitochondria were fragmented in most MFN2- cells (**Fig. 5B**). In a blinded review, nearly 70% of mitochondria in CRE-treated cells appeared fragmented vs. 10–15% in either control (*P*<0.05; **Fig. 5C**).

### MFN2-deficiency increases susceptibility to apoptosis following metabolic stress

**Figure 6:**
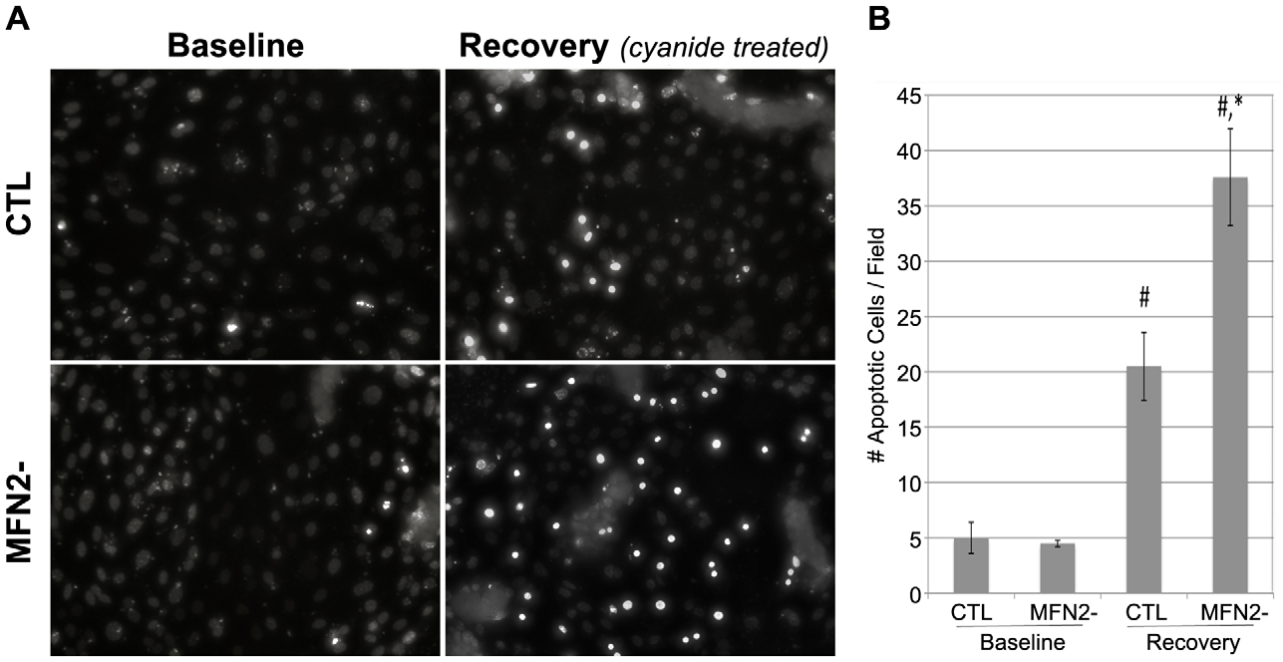
Effect of MFN2 deficiency on apoptosis after metabolic stress. (**A**) Wide field fluorescent microscopy of Cre (MFN2-) and empty (CTL) adenovirus transduced cells stained with Hoechst dye after 3 hr ATP depletion induced by cyanide exposure in glucose free medium followed by and 6 hr recovery in complete medium; apoptotic cells are brightly stained, small and round. (**B**) Quantitative analysis of apoptotic cell number in five randomly selected fields under each experimental condition; ^#^ *P*<0.05 *vs.* EV/Base or CRE/Base, respectively; * *P*<0.05 vs. EV/Rec. Data represent the mean and SE of at least 3 separate experiments.

In the absence of glucose in the cell culture medium, exposure of cells to metabolic inhibitors such as cyanide or rotenone causes apoptosis in proximal tubule cells [40], [41], [42]. MFN2^f/f^ cells were transduced with either an empty or CRE-containing adenovirus producing MFN2 replete (CTL) or MFN2 deficient (MFN2-) cells respectively. Subsequently, CTL and MFN2- cells were stress by three hours of cyanide exposure followed by six hours recovery in glucose containing medium. In the absence of stress (*baseline*), minimal apoptosis was detected in Hoechst stained cells and this was not influenced by MFN2 deficiency (**Fig. 6A**, *left panels*). During recovery from stress (*recovery*) however, the number of apoptotic cells was far greater in MFN2 deficient cells (Fig. 6A, *lower right panel*) compared to control (Fig. 6A, *upper right panel*). In at least five randomly selected fields, a blinded observer detected an 83% increase in apoptosis in MFN2 deficient cells compared to control (**Fig. 6B**).

### MFN2 deficiency in renal proximal tubule cells does not alter the oxygen consumption profile or Bax 6A7 epitope exposure

**Figure 7:**
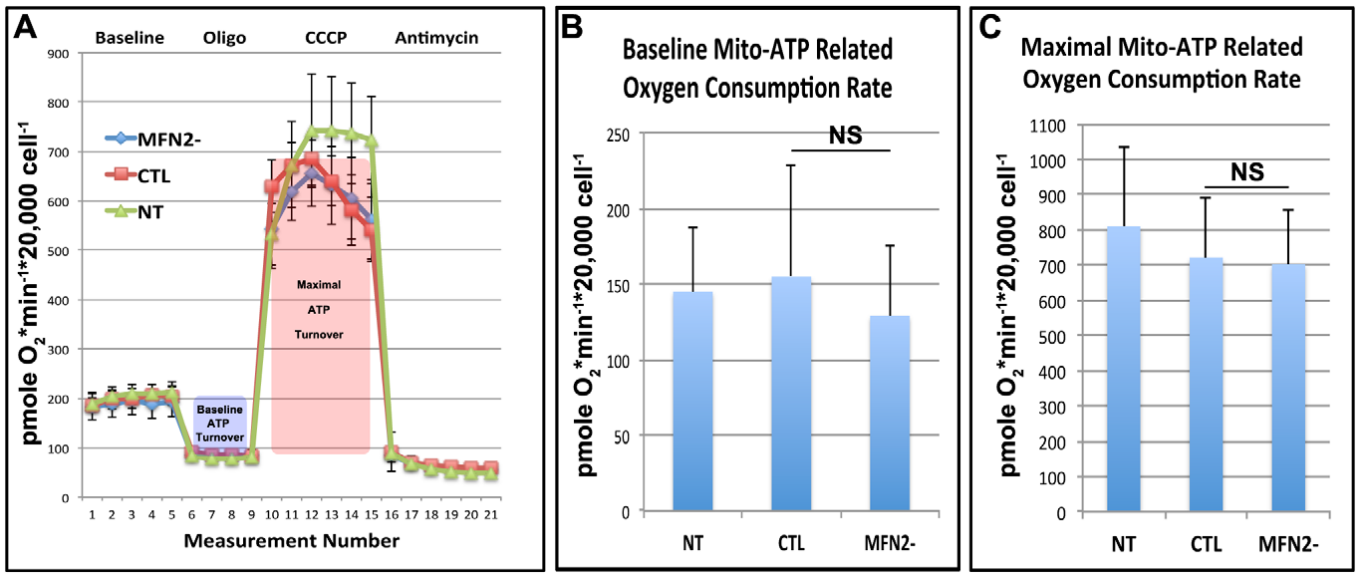
Effect of MFN2 deficiency on oxygen consumption rate (OCR) profile. (**A**) Serial measurements of OCR at 7 minute intervals (“measurement number”) in MFN2-deficienct (MFN2-) and control (empty virus transduced *“CTL”* or non-transduced; *“NT”*) cells at baseline and after the addition of oligomyin, CCCP, or antimycin. Results of a single representative study with five replicate OCR measurements and their SD is shown. (**B**) OCR due to baseline ATP turnover rate (*baseline minus oligomycin OCR*) in each group expressed as the mean and SD of 3 separate experiments; NS “non significant; *P*>0.05. (**C**) OCR due to maximal mitochondrial ATP turnover rate (*CCCP stimulated minus oligomycin OCR*) in each group expressed as the mean and SD of 3 separate experiments; NS “non significant; *P*>0.05.

Despite normal kidney and tubule function, proximal tubule epithelial cells with reduced MFN2 content are more susceptible to stress. To determine whether this susceptibility is due to altered mitochondrial metabolism, oxygen consumption rate (OCR) was measured in control and MFN2 deficient cells at baseline as well as in the presence of oligomycin (an oxidative phosphorylation inhibitor), CCCP (a mitochondrial uncoupler), or antimycin (an electron transport inhibitor). The OCR profile was similar in non-treated (NT), CTL, and MFN2- cells (**Fig. 7A**). The OCR related to baseline ATP turnover rate (baseline minus oligomycin OCR) and maximal mitochondrial ATP turnover rate (CCCP-stimulated minus oligomycin OCR) were calculated in three separate experiments. Despite marked differences in morphology, both baseline ATP turnover (Fig. 7B; *P* = 0.6) and maximal mitochondrial ATP turnover OCR (Fig. 7C; *P* = 0.9) were indistinguishable in CTL and MFN2- cells. Within 10 minutes, exposure of CTL or MFN2- renal epithelial cells to sodium cyanide resulted in a similar, profound and sustained decrease in OCR (*data not shown*).

**Figure 8:**
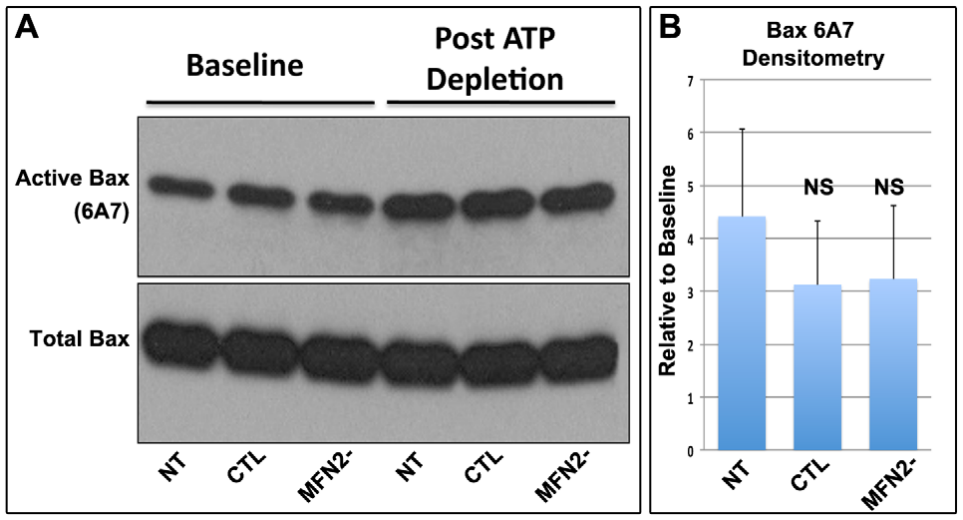
Effect of MFN2 deficiency on Bax activation. (**A**) Immunoblot of active Bax (*upper panel*) detected with an anti-6A7 epitope-specific antibody in MFN2^f/f^ proximal tubule epithelial lysates of cells transduced with no virus (*NT*), empty adenovirus (*CTL*) or Cre adenovirus (*MFN2-*) at baseline and 30 min after ATP depletion; total Bax controls are show for sample loading *(lower panel)*; and (**B**) densitometric analysis of the relative increase in active 6A7-Bax content after metabolic stress.

Bax is a critical pro-apoptotic protein responsible for outer mitochondria membrane injury after metabolic stress [29], [32], [38], [43]. To determine whether MFN2-deficiency enhanced Bax activation, we measured Bax 6A7 epitope exposure in lysates harvested from MFN2 deficient (MFN2-) and CTL renal proximal tubule epithelial cells before and after APT depletion. ATP depletion caused a comparable increase in Bax 6A7 exposure regardless of the level of MFN2 expression (**Fig. 8A and B**; *upper panel*). Importantly, total Bax content was similar in all groups under all experimental conditions (*lower panel*).

### MFN2-deficiency promotes Bax translocation to mitochondria following metabolic stress

**Figure 9:**
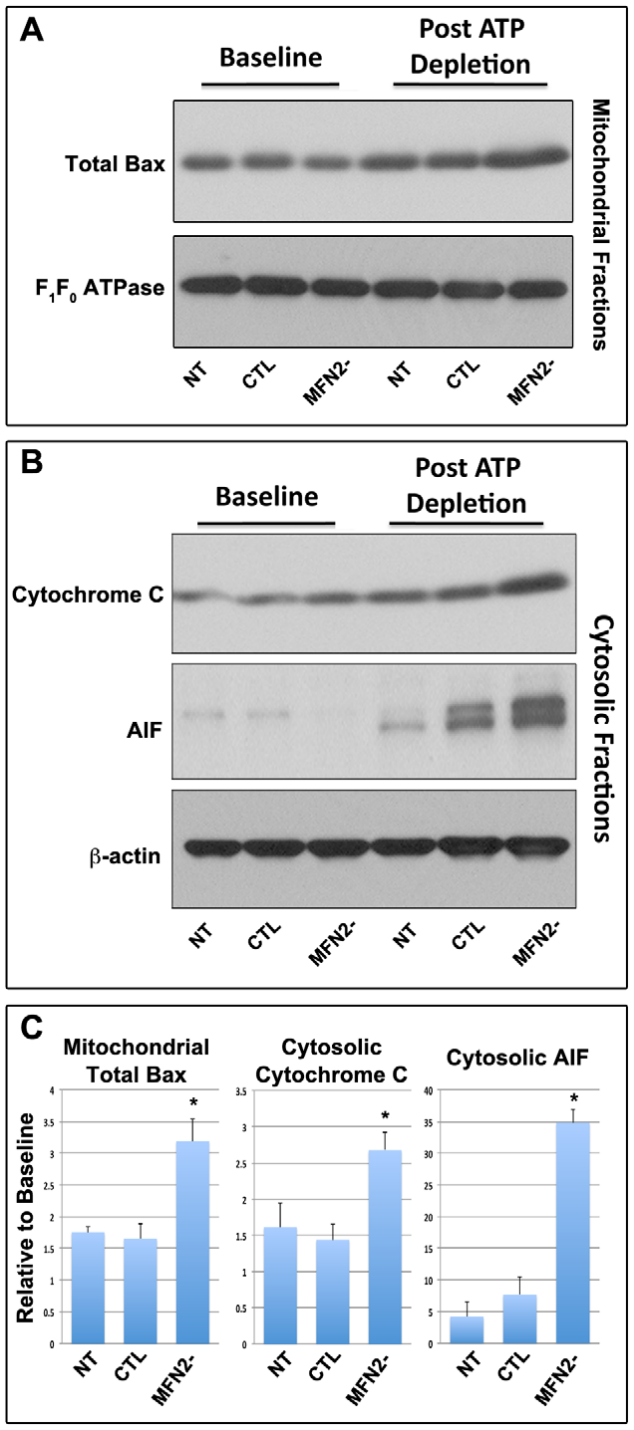
Effect of MFN2 deficiency on mitochondrial Bax translocation, and outer membrane injury. (**A**) Immunoblot of Bax (*upper panel*) in mitochondrial membrane fractions of MFN2^f/f^ proximal tubule epithelial cells exposed to no virus (*NT*), empty virus (*CTL*) or Cre adenovirus (*MFN2-*) at baseline and 30 min post ATP-depletion; F_1_F_0_ ATPase serves as loading control (*lower panel*). (**B**) Immunoblot analysis of cytochrome c (*upper panel*) and Apoptosis Inducing Factor (AIF) (*middle panel*) in cytosolic fractions harvested from non-viral transduced cells (*NT*) or cells transduced with either empty virus (*CTL*) or Cre adenovirus (*MFN2-*) at baseline and 30 min after ATP depletion. ß-actin loading control (*lower panel*). (**C**) Densitometric analysis of mitochondrial Bax accumulation as well as the leakage of cytochrome c and AIF in renal tubule epithelial cells after ATP depletion; data represent the mean and SE of 4 separate experiments; *P*<0.05.

To test the hypothesis that fragmented mitochondria in MFN2- cells may be more susceptible Bax-mediated injury, Bax accumulation and outer membrane injury were assessed in isolated mitochondria before and after ATP depletion. Relatively low levels of Bax were detected at baseline in mitochondria isolated from CTL and MFN2- cells. After stress however, mitochondria isolated from MFN2- cells exhibited significantly greater mitochondrial Bax content than CTL cells (**Fig. 9A and 9C**). Furthermore, Bax accumulation in MFN2- cells increased outer member injury and promoted apoptosis, the release of cytochrome c and Apoptosis Inducing Factor (AIF), pro-apoptotic proteins normally restricted to the inter-mitochondrial membrane space (**Fig. 9B & 9C**). As expected, cytosolic cytochrome c levels were relatively low in partially permeabilized, non-transduced proximal tubule cells at baseline. At baseline, cells transduced with either EV or CRE showed slight (but comparable) leakage of mitochondrial cytochrome c compared to non-transduced cells (**Fig. 9B**). In contrast, MFN2- proximal tubule cells released significantly more cytochrome c than CTL cells after ATP depletion (**Fig. 9B**). Similarly, AIF leakage (**Fig. 9B**) was more pronounced in MFN2- vs. CTL cells, indicating that the lack of MFN2 increases the susceptibility to outer mitochondrial membrane injury following stress. Densitometric analysis of these data confirmed that MFN2-deficiency increased mitochondrial Bax accumulation that was associated with increased outer membrane injury (**Fig. 9C**).

## Discussion

Prior reports demonstrate that mitochondrial re-modeling is crucial for normal development and function in some mammalian tissues (e.g., neuronal, cardiac and pancreatic development) [11], [13], [44], [45] and that dynamic mitochondrial fusion and fragmentation is necessary to maintain a healthy mitochondrial population with optimal aerobic ATP production capacity [2], [3], [4], [11], [46]. Despite these data in other tissues, we report here that MFN2-mediated mitochondrial fusion is not necessary for kidney development, tubule function during the perinatal period, and normal ATP synthesis in proximal tubule epithelial cells. MFN2-deficient proximal tubule cells had fragmented mitochondria (Fig. 5C) but nevertheless appear healthy, proliferate normally and exhibit normal baseline and maximal mitochondrial ATP turnover as measured by OCR (Fig. 7). Similarly, renal glomerular filtration is normal on postnatal day 4 (fig. 4B) and tubular function (i.e., reabsorption of filtered protein or glucose) is unaffected by MFN2-deficiency. Even though the kidneys of MFN2 cKO mice have 20% fewer nephrons (Fig. 2B), this relatively small decrease in nephron number would not be expected to adversely affect organ function [47]. Although mitochondria are abundant in the kidney [48], [49], large-scale organelle fragmentation does not alter renal structure or impair its function.

Despite apparently normal renal function, MFN2 cKO mice die within 6 days of birth. Although the cause of death in MFN2 deficient animals is uncertain, they are underweight, lack milk spots (a sign of poor feeding) and exhibit an unsteady gait. A similar phenotype has been described in mice lacking MFN2 in the cerebellum [14] and, as the Pax2 promoter selected in the present study is also expressed in the mid- and hind-brain [50], extra-renal loop-out of the MFN2^f^ allele in the central nervous system is likely the cause of the early death in our mice. Although Pax2-Cre^+^/MFN2^f/+^ mice survived and exhibited intermediate body weight, highly variable mitochondrial morphology was observed, precluding further studies in Pax2-Cre^+^/MFN2^f/+^ mice or cells harvested from their kidneys. As a result, our *in vivo* model of MFN2-deficiency is limited as we are unable to analyze the role of MFN2 in mature collecting duct cells that is only seen in three to four week old mice. We are therefore unable to study the susceptibility of MFN2 cKO to acute or chronic kidney disease. The small blood volume collected from four-day old pups also precludes measurement of creatinine, a second estimate of GFR that could confirm our BUN data. We recognize that MFN1 and MFN2 may have partially redundant functions and while the marked fragmentation observed in MFN2-deficient cells both *in vivo* and *in vitro* may suggest that MFN2 is the major mediator of mitochondrial fusion in renal epithelial cells, we have not addressed the role of MFN1 in our studies.

The kidney is somewhat unique in that it operates in a relatively hypoxic environment and is therefore remarkably susceptible to ischemic injury [51]. Mitochondrial fragmentation and fusion are recently reported to be involved in both acute and chronic cellular stress responses in the kidney. Renal ischemia-reperfusion injury *in vivo* causes a marked shift from filamentous to fragmented mitochondria [39] and mice treated with a Drp1 inhibitor, which prevents fragmentation, are protected from ischemia- injury [39]. Similarly, MFN2 over-expression that promoted mitochondrial fusion has been suggested to delay the onset of chronic diabetic nephropathy in mice [52]. In keeping with these *in vivo* data, Hela cells over-expressing MFN1 or MFN2, as well as rat proximal tubule cells expressing dominant negative Drp1, have filamentous mitochondria and are protected from azide or cisplatin-induced apoptosis [39]. In contrast, Mouse embryonic fibroblasts from MFN1 or 2 knockout mice have increased mitochondrial fragmentation and are more prone to injury-induced cell death [39]. In the kidney, we show that mouse proximal tubule cells, a primary target of acute and chronic renal injury, are more susceptible to metabolic stress when MFN2 expression is reduced and mitochondria fragmented. MFN2-deficiency did not affect cell survival at baseline or mitochondrial energetics (Fig. 7) but markedly increases the leakage of both cytochrome c and AIF from the outer mitochondrial membrane following ATP depletion (Figs. 6 and 9), demonstrating that MFN2 plays an important role in protecting renal tubule cells from stress-induced apoptosis. Our data suggest that targeting mitochondrial dynamics may be an important therapeutic option to ameliorate tubular cell death following acute or chronic renal insults.

How fission and fusion mediate susceptibility to renal cell death is presently unclear. Given that BCL proteins regulate mitochondrial injury during *in vitro* metabolic stress [30] and following ischemia-reperfusion injury *in vivo* [31], proteins in this family are “primary suspects”. Stress-induced changes in the balance between pro-apoptotic (e.g., Bax or Bak) and anti-apoptotic (e.g., Bcl-2, Bcl-xl) BCL family proteins cause outer mitochondrial membrane injury and cell death. In the proximal tubule, Bax plays a central role in organelle injury and death [28], [31], [33], [53], [54]. “Bax attack” of the outer mitochondrial membrane requires multiple steps for optimal activation, thereby creating several checkpoints in the apoptotic death pathway. These steps include exposure of the Bax N-terminal 6A7 epitope, translocation to the outer mitochondrial membrane, oligomerization, clustering, and finally, membrane insertion [55], [56], [57], [58], [59]. In the proximal tubule cell, metabolic stress such as ATP depletion exposes the 6A7 epitope [29], [31], [32], [38], [43], causes Bax oligomerization [29], [43], mitochondrial Bax accumulation [29], [31], [38] and insertion into mitochondrial membrane [38], leakage of pro-apoptotic molecules [29], [30], [39], [60] and apoptosis [31], [32], [39]. Importantly, inhibiting Bax expression or over-expressing Bcl2 both reduce mitochondrial injury in our model (*data not shown*) as well in other models of metabolic stress [33], [39]. Inactive Bax may promote mitochondrial fusion in healthy neuronal cells [25], suggesting that it plays opposing roles in both health and disease.

Recent work by Brooks, et. al., shows that mitochondrial re-modeling and Bax may be linked [38]. These investigators reported that organelle fission enhances Bax toxicity, whereas pro-fusion events have the opposite effect and protect the outer membrane from injury caused by cisplatin or metabolic stress [38]. *In non-renal cells*, mitochondrial elongation (MFN1, MFN2 or dominant negative DRP-1 expression) and fragmentation (MFN 1 or 2 knockout) had opposing effects on Bax 6A7 epitope exposure (often viewed as upstream of changes in organelle morphology), Bax oligomerization, and membrane insertion but had no consistent effect on mitochondrial Bax accumulation [38] required for outer membrane injury [61]. In contrast, we now show that MFN2 deficient renal proximal tubule cells subjected to stress exhibit equivalent 6A7 epitope exposure compared to control (fig. 8) but are significantly more susceptible to Bax accumulation (fig. 9A), outer membrane injury (fig. 9B,C) and apoptosis (fig. 6). These results suggest that Bax 6A7 epitope exposure occurs primarily in the cytosol of renal cells and is relatively unaffected by downstream mitochondrial membrane events. In our model, organelle re-modeling appears to alter mitochondrial membranes in a manner that promotes Bax accumulation, possibly by exposing as yet undefined binding sites or altering their affinity of mitochondria for active Bax.

In summary, we propose that mitochondrial re-modeling and particularly MFN2, play a unique role in the renal stress response. Despite abnormal mitochondrial morphology, the MFN2-deficient kidney exhibits normal development, filtration and tubular function. In contrast, mitochondrial fragmentation caused by MFN2 deficiency markedly increases the susceptibility of the organelle to “Bax attack” during metabolic stress, perhaps by altering membrane properties to facilitate Bax insertion and pore formation. Future studies will define the relationship between mitochondrial membrane re-modeling and BCL family proteins that mediate apoptosis and organ dysfunction following ischemic injury *in vivo*.

## Methods

### Ethics Statement

All animals were maintained under the guidance of the Boston University animal core facility following NIH and IACUC guidelines. Animal work was carried out under protocol number AN-10524 entitled “Mechanisms of Acute Renal Failure.” The protocol is APPROVED by the IACUC at Boston University Medical Center as being consistent with humane treatment of laboratory animals and with standards set forth in the Guide for the Care and Use of Laboratory Animals and the Animal Welfare Act. Boston University Medical Center has had an Animal Welfare Assurance on file with the Office of Laboratory Welfare (OLAW) since January 1, 1986. The Animal Welfare Assurance number is A-3316-01. The Laboratory Animal Science Center at Boston University Medical Center has been accredited by the American Association for Accreditation of Laboratory Animal Care (AAALAC) since 1971.

### Animals

Mitofusin 2 floxed animals (MFN2^f/f^) were originally generated by the Chan laboratory [8]. Conditional kidney knockouts were created by breeding MFN2^f/f^ animals with animals expressing Cre recombinase under the control of the Pax2 promoter (Pax2-Cre, MMRRC Stock Tg(Pax2-Cre)1Akg [50]).

### Primary cell culture

As previously described [62], [63]. Briefly, three-week old mice were sacrificed, their kidneys removed, and cortex harvested. The cortex was minced, placed into HBSS containing collagenase IV (1 mg/mL), and incubated for 60 min at 37°C. The collagenase was neutralized by addition of fetal calf serum, washed once in red cell lysis buffer (Sigma, St. Louis, MO) and plated into culture dishes in selection medium containing DMEM and Ham's F12 medium (50∶50 vol∶vol) and a mixture of insulin (5 mg/L), hydrocortisone (50 nM), apotransferrin (500 mg/L) and penicillin/streptomycin. Cultures were incubated for 5–7 days in 5% CO_2_ and at 37°C and the cells were characterized as proximal tubule cells [62], [63].

### Metabolic stress

ATP depletion is an established model of renal ischemia that sustains ATP content at <10% baseline values until recovery is initiated [40], [41], [42], [64]. To initiate ATP depletion, cells were washed 3 times in glucose-free DMEM (Invitrogen, Carlsbad, CA; L-glutamine 584 mg/L, without pyruvate) followed by incubation in glucose-free DMEM containing sodium cyanide (5 mM) and 2-deoxy-D-glucose (5 mM) for 1–3 hours. Recovery was initiated by replacing the above media with complete primary cell culture media.

### *In vitro* recombination of MFN2 floxed alleles by Cre recombinase

An adenovirus expressing Cre recombinase was used to transduce MFN2^f/f^ primary proximal tubule cells. The cells were infected with adenovirus (10 m.o.i.) overnight and the medium was replaced with complete primary culture media for 48 hr prior to the experiments. Recombination of the loxP sites by Cre recombinase deleted the floxed MFN2 gene creating MFN2 deficient cells in culture. Adenovirus without cre was used as control.

### Antibodies

The MFN2 (XX-1) and AIF antibodies were purchased from Santa Cruz Biotechnology (Santa Cruz, CA). The F_1_F_0_ ATPase (complex V ß subunit) Antibody was purchased from Invitrogen (Carlsbad, CA). Cytochrome c antibody was purchased from Clontech (Mountain View, CA). Active-Bax (6A7) antibody was purchased from Trevigen (Gaithersburg, MD). Total Bax antibody was purchased from Cell Signaling Technologies (Danvers, MA). The appropriate secondary antibody conjugated to AlexFluor-488 (Invitrogen) for immunofluorescence or to horseradish peroxidase (Bio-Rad) was used for immunoblotting.

### Immunoblot analysis

Cell proteins were extracted with NP-40 buffer (50 mM Tris HCl, pH 8.0; 150 mM NaCl; 0.4% NP-40) containing a protease inhibitor cocktail (Set I; Calbiochem, San Diego, CA). Samples were sonicated, spun at 10,000× g, and supernatants collected. Subcellular fractions were obtained as previously described [65]. Briefly, cells were harvested in isotonic mitochondrial buffer (210 mM mannitol, 70 mM sucrose, 1 mM EDTA, and 10 mM Hepes pH 7.5) containing protease inhibitor and homogenized for 40 strokes with a dounce homogenizer. Lysates were centrifuged at 500× g for 5 min to remove unbroken cells and nuclei. The supernatants were centrifuged at 10,000× g for 30 min to pellet the membrane fraction. The resulting supernatant was stored as cytosol. The membrane fraction pellets were solubilized in RIPA buffer containing EDTA (Boston BioProducts) and vortexed for 2 min. After freezing and thawing on ice to ensure release of membrane bound proteins, the membrane fractions were centrifuged at 10,000× g for 10 min and the supernatant saved as the mitochondrial fraction. Proteins from kidney tissue homogenates were extracted in NP-40 buffer by homogenization using an Ultra-Turrax disperser (IKA, Wilmington, NC) followed by sonification and centrifugation as above. Protein levels were measured using the BCA assay (Pierce) and equal amounts of protein (10–20 µg) were separated on 7.5–12% tris-glycine polyacrylamide gels. Separated proteins were transferred to nitrocellulose membranes and antigens were detected using specific primary antibodies.

### Oxygen Consumption Rate (OCR)

Primary cell cultures were transferred to Seahorse XF24 plates. OCR was measured the following day on the XF24 flux analyzer (Seahorse Biosciences, Billerica, MA). Five replicate OCR measurements were obtained at baseline and following injection of oligomycin (5 µM), CCCP (40 µM) and antimycin (10 µM) and were normalized to cell number. Cells were fixed in 4% paraformaldehyde for 10 min, washed with PBS and incubated in PBS containing Hoechst (5 µg/mL for 30 min) before being imaged and counted in an automated cell counter (Celigo Adherent Cell Cytometer; Cyntellect, San Diego, CA). OCR due to baseline and maximal mitochondrial ATP turnover were calculated as described in the Seahorse Operator's Manual.

### Histology and Fluorescence

#### *In vitro*

For mitochondrial imaging, cells were cultured in MatTek 35 mm dishes (MatTek Corp, Ashland, MA), stained with MitoTracker Green FM (200 nM; Invitrogen, Carlsbad, CA) for 1 hour, and imaged by confocal microscopy (Perkin Elmer Ultraview Spinning Disc, Wellesley, MA). For Hoechst staining, cells were stained for 30 min in Hoechst dye #33342 (5 mcg/mL) and imaged by wide-field fluorescence microscopy (Nikon Deconvolution wide field epifluorescence microscope, Melville, NY).

#### *In vivo*

Kidneys were removed from 4-day-old pups and placed in 4% paraformaldehyde overnight at 4°C. Following 2 washes in PBS the tissue was placed in 15% sucrose for 1 hour followed by 30% sucrose overnight. The tissue was then embedded in Tissue-Tek O.C.T compound and cut into 5 mm sections using a cryostat. To stain mitochondria, sections were washed 3 times for 5 min each in PBS followed by 5 min in PBS containing 0.1% SDS. After 3 additional washes, tissue sections were blocked in PBS containing 5% normal goat serum and 0.3% Triton X-100 for 1 hr. Following 3 washes, sections were incubated with antibody against F_1_F_0_ ATPase (2 mg/ml) in PBS with 1% BSA and 0.3% Triton X-100 for 90 min. After 3 PBS washes, sections were incubated in secondary antibody conjugated AlexaFluor 488 (1∶250 dilution in 1% BSA, 0.3% Triton X-100 in PBS) for 1 hr, washed 3 final times in PBS, mounted with gelvatol containing Hoechst and examined by confocal microscopy. For staining MFN2 sections were washed 3 times in PBS for 5 min followed by microwaving on high in 10 mM sodium citrate pH 5.5 for 15 min. Sections were incubated with MFN2 primary antibody (1∶50 dilution) then performed as above for F_1_F_0_ staining. To measure apoptosis, intraperitoneal injection of Hoechst dye #33342 (100 µg in 100 µl) was performed prior to sacrifice. One hour post-injection, the animals were sacrificed, and the kidneys were fixed with paraformaldehyde and sectioned as above. For renal histology, fixed kidneys were embedded in paraffin, sectioned with a microtome, and counter stained with hematoxylin and eosin (BU Pathology Core, Boston, MA). Randomly selected tissue sections were analyzed for the number of apoptotic cells by a blinded observer. Glomerular number, cortical thickness and nephrogenic zones were analyzed in a similar fashion using established criteria [66].

### BUN and Hematocrit

On day 4 after birth, mice whole blood was collected from the heart in capillary tubes spun in a hematocrit centrifuge. Serum was collected from the tubes and BUN measured using a QuantiChrom BUN assay kit (Bioassay Systems, Hayward, CA) following the manufacturer's protocol.

### Densitometry

After digitizing each immunoblot image (Hewlett-Packard, Desk Scan II), selected band densities were quantified using NIH Image J Software. Data are expressed as the mean ± SE.

### Statistical Analysis

Data were analyzed using the Excel (Microsoft, Redmond, WA). Significant differences between groups were determined by an unpaired Student's t-Test with values of *P*<0.05 considered as significant.
